# Supplementary material for: Differential Chemical Profile of Metabolite Extracts Produced by the Diaporthe citri (G-01) Endophyte Mediated by Varying the Fermented Broth pH
Source: Metabolites. 2022 Jul 26;12(8):692. doi: 10.3390/metabo12080692 (PMC9330126; doi:10.3390/metabo12080692)
Supplement: Supplementary file 1 [file metabolites-12-00692-s001.zip › metabolites-1780485-supplementary.pdf]

Differential Chemical Profile of Metabolite Extracts Produced by the *Diaporthe citri* (G-01) Endophyte Mediated by Varying the Fermented Broth pH

- Supplementary material -

Normalization and multivariate statistic approaches.

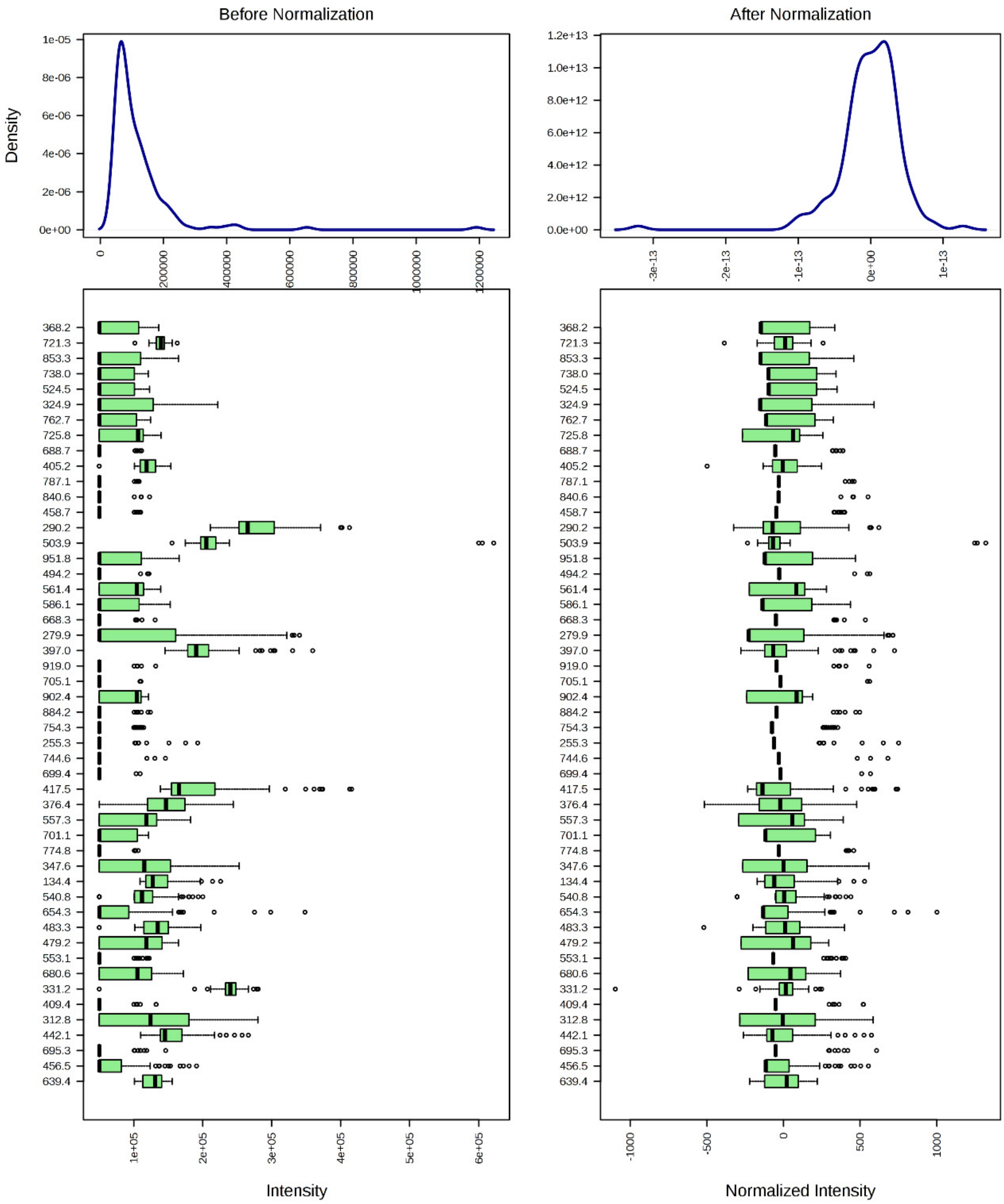

Figure S1. Pareto scaling normalization of the features acquired in positive mode.

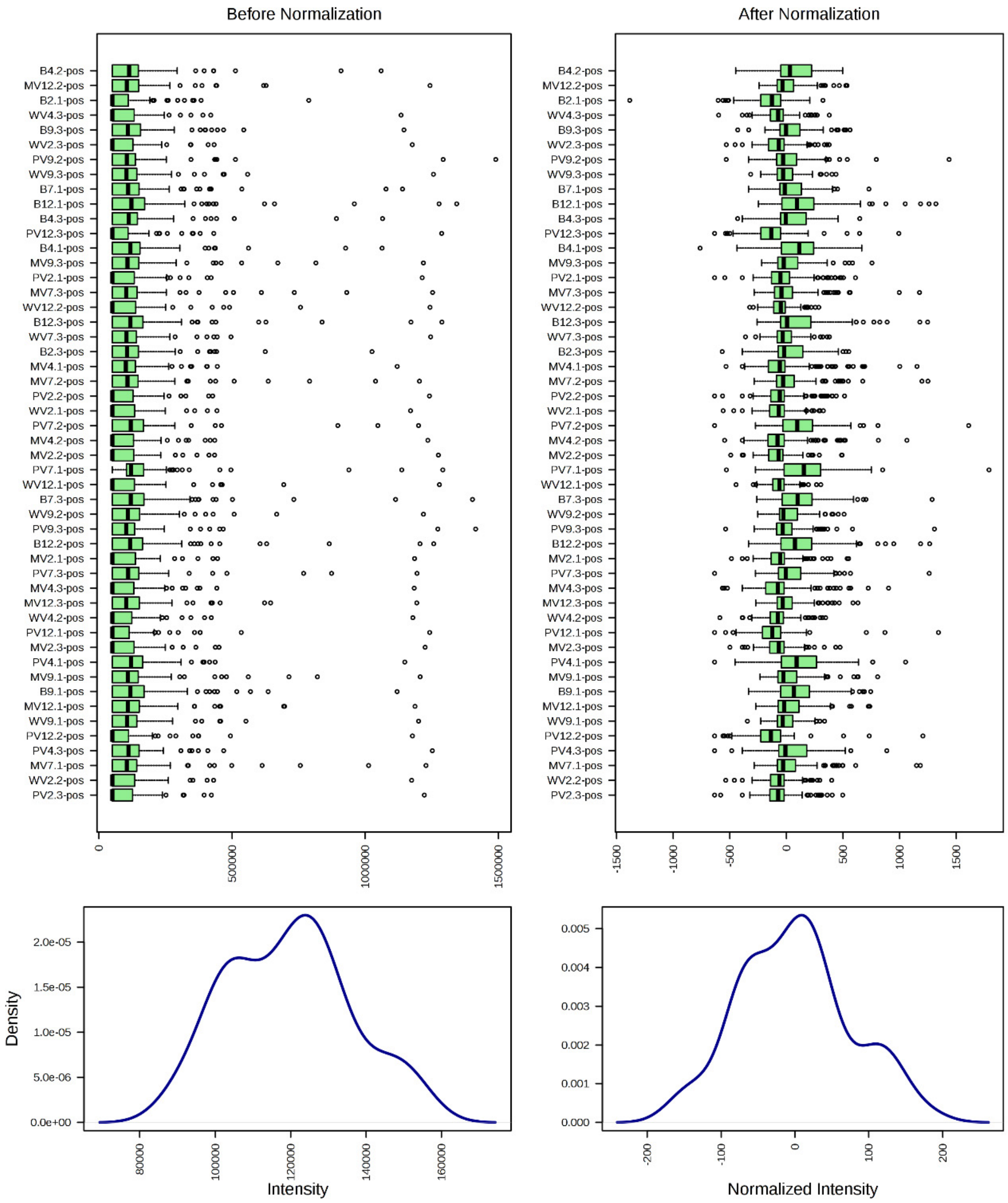

Figure S2. Pareto scaling normalization of samples acquired in positive mode.

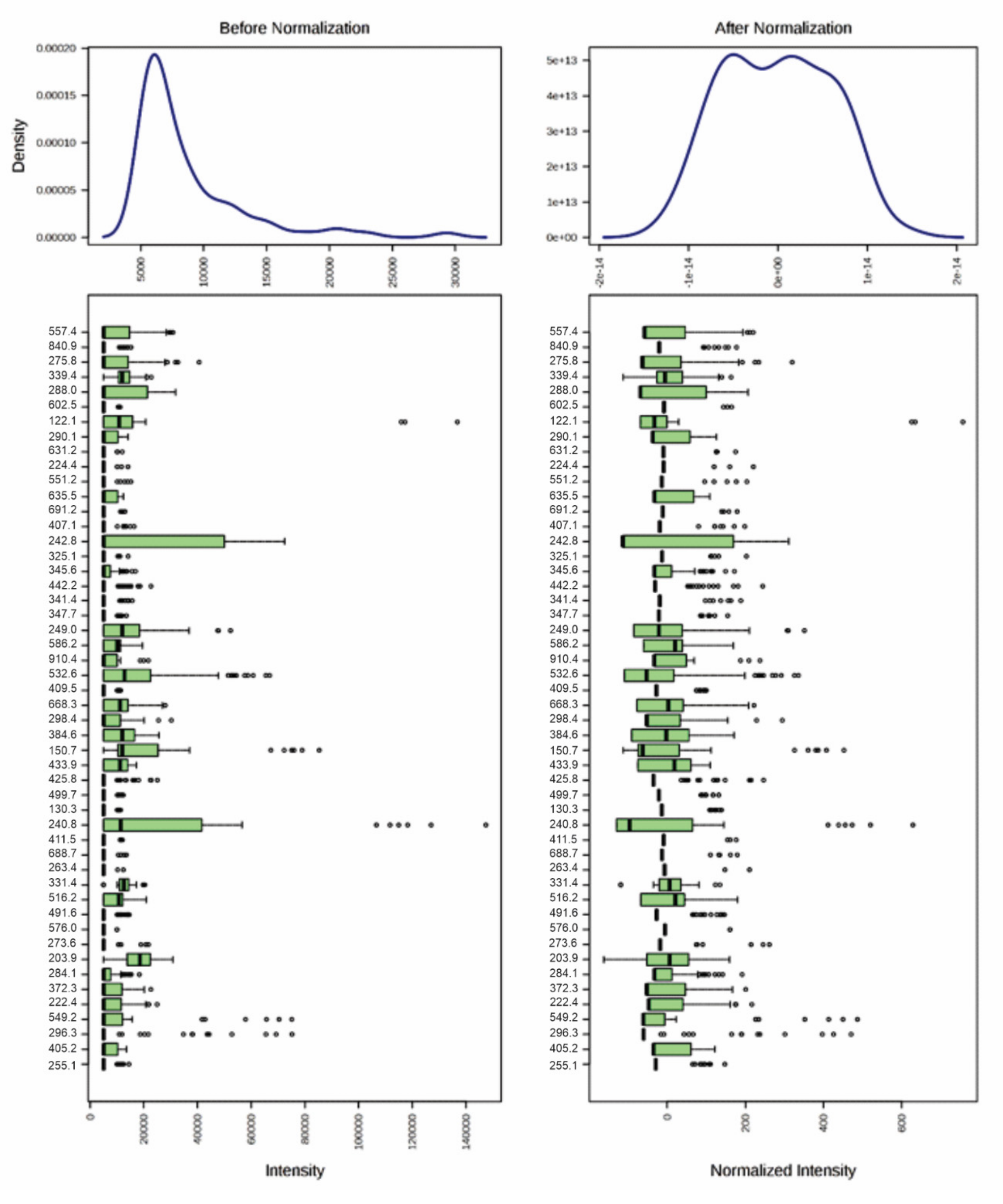

Figure S3. Pareto scaling normalization of features acquired in negative mode.

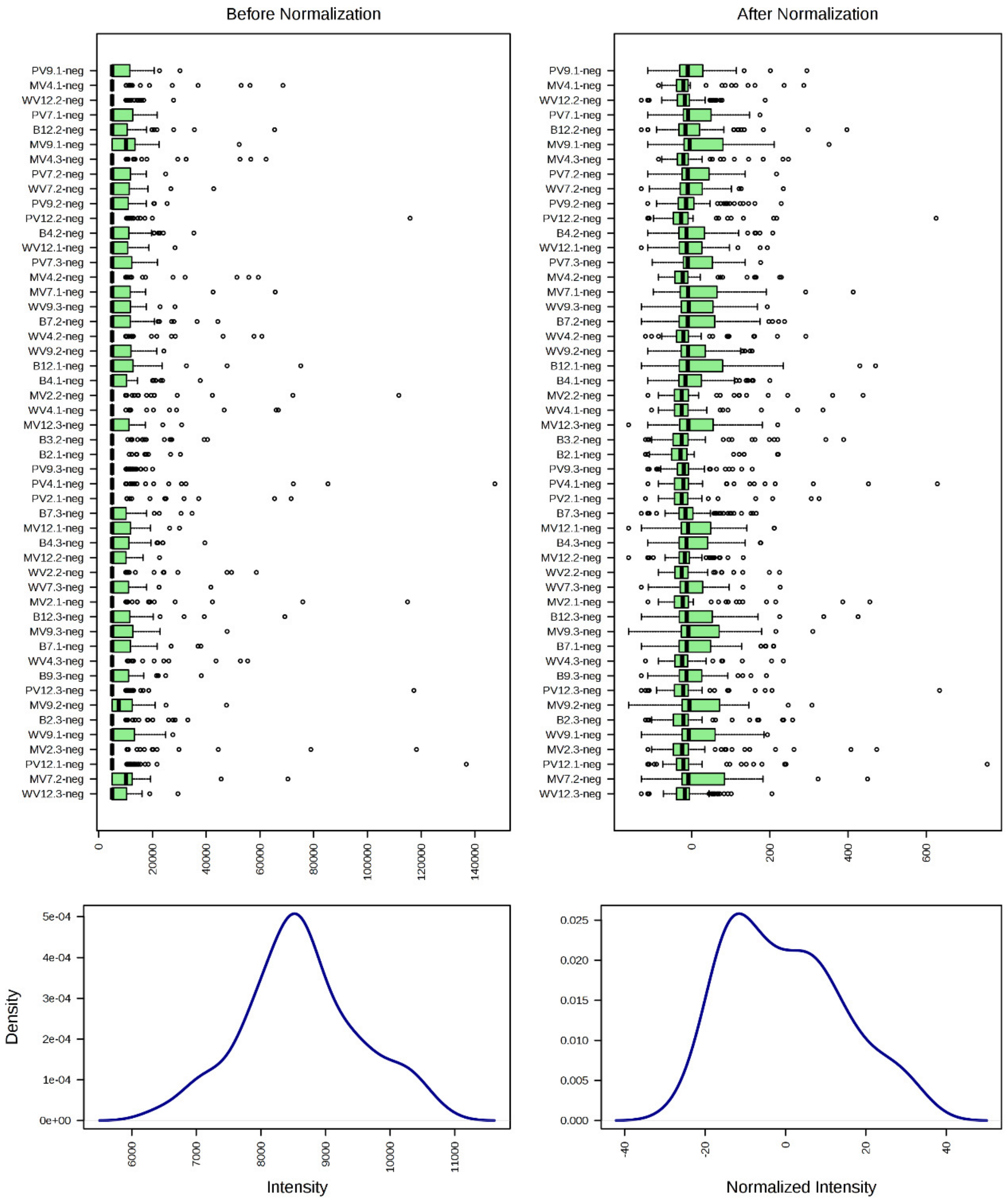

Figure S4. Pareto scaling normalization of samples acquired in negative mode.

Results of Multivariate analyses

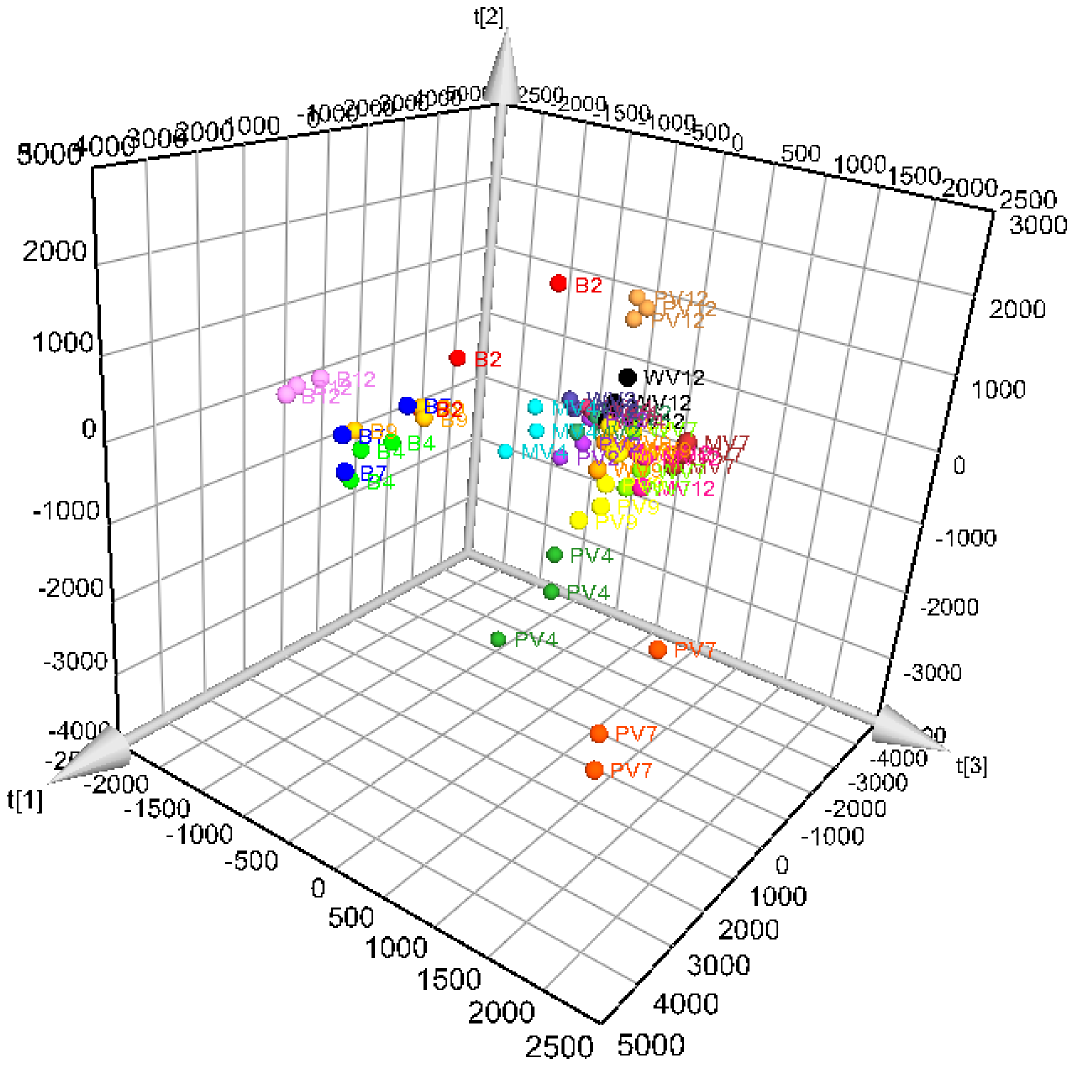

Figure S5. First three principal components to positive data: PC1 (31.9%), PC2 (11.7%), and PC3 (10.8%).

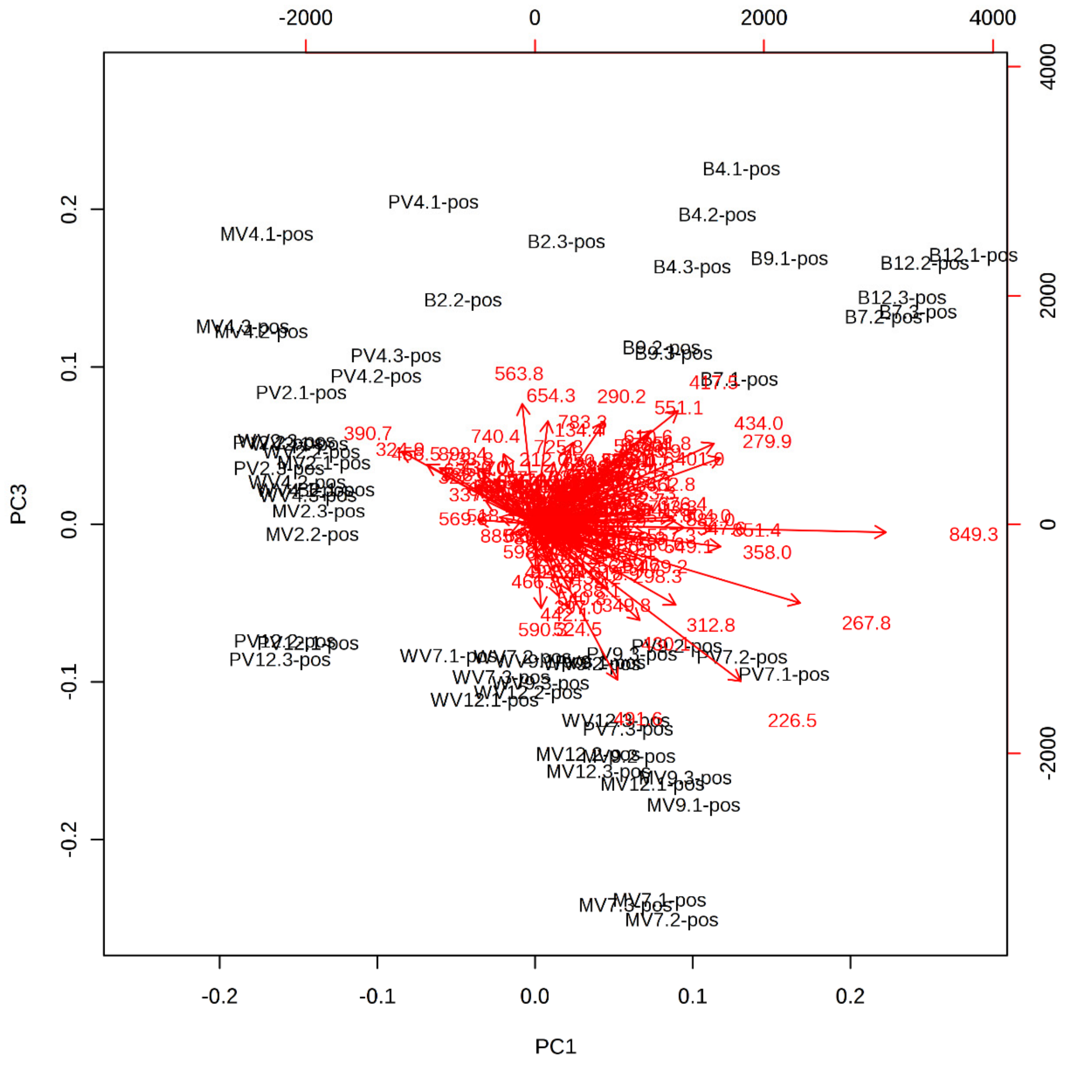

Figure S6. Bi-plot analysis of positive mode acquired data for PC1 (31.9%) and PC3 (10.8%).

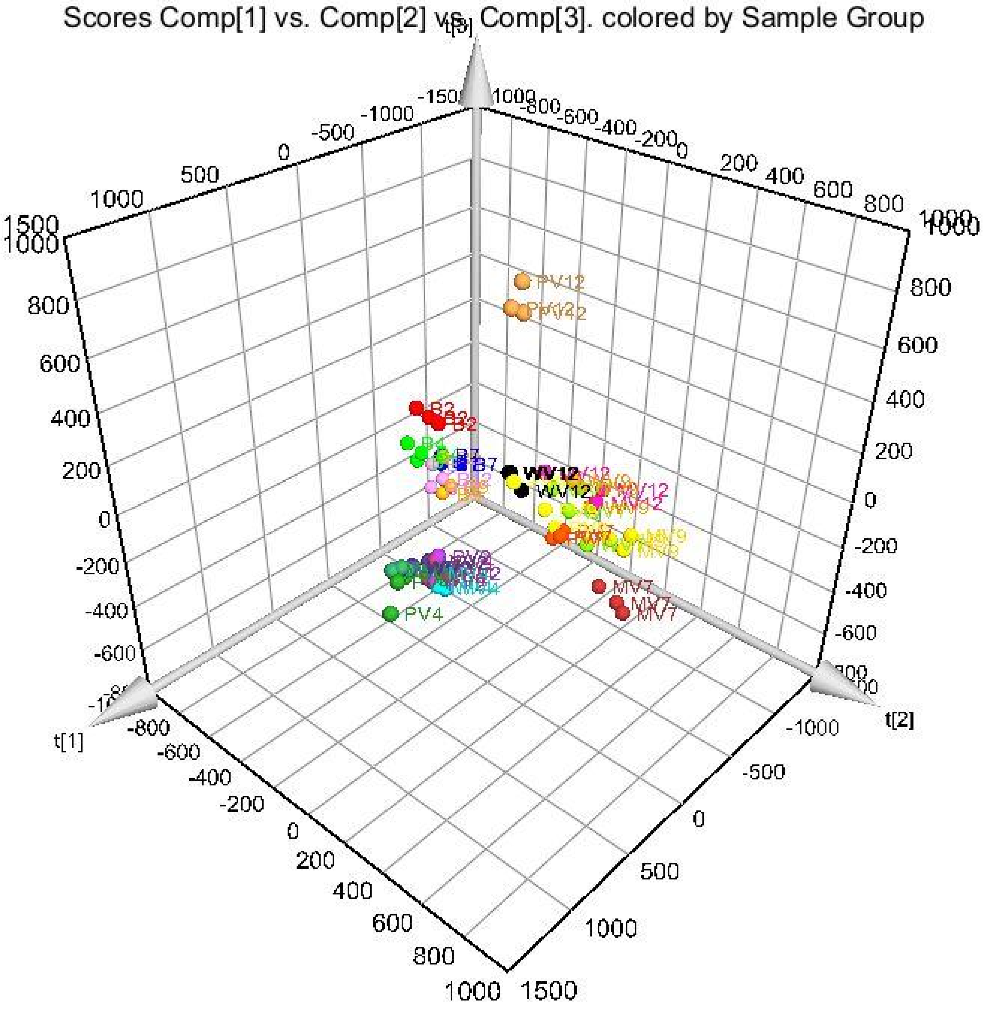

Figure S7. First three principal components to negative data: PC1 (32.8%), PC2 (14.2%), and PC3 (9.4%).

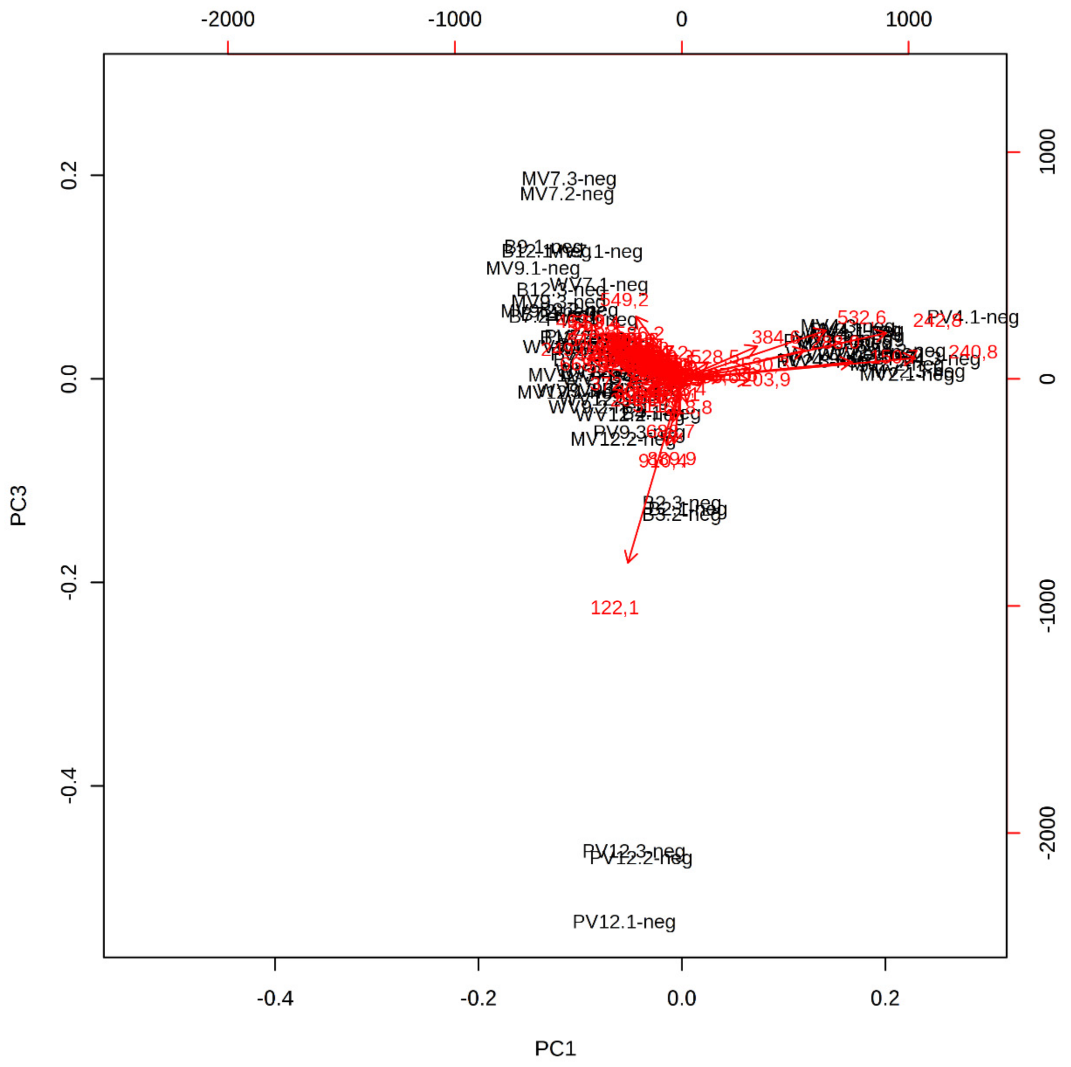

Figura S8. Bi-plot analysis of negative mode acquired data for PC1 (32.8%) and PC3 (9.4%).

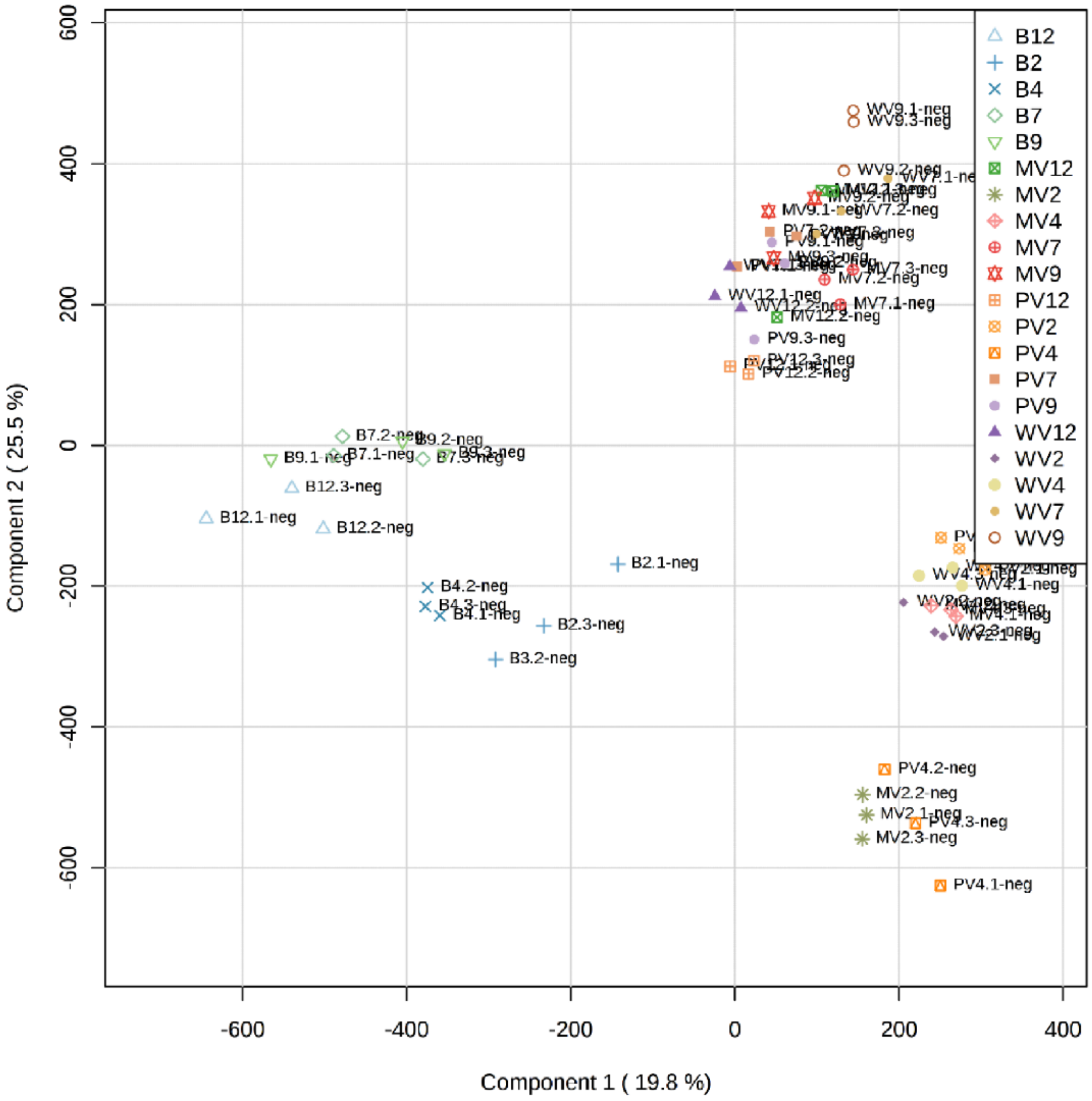

Figure S9. PLS-DA analysis of samples obtained in negative mode.

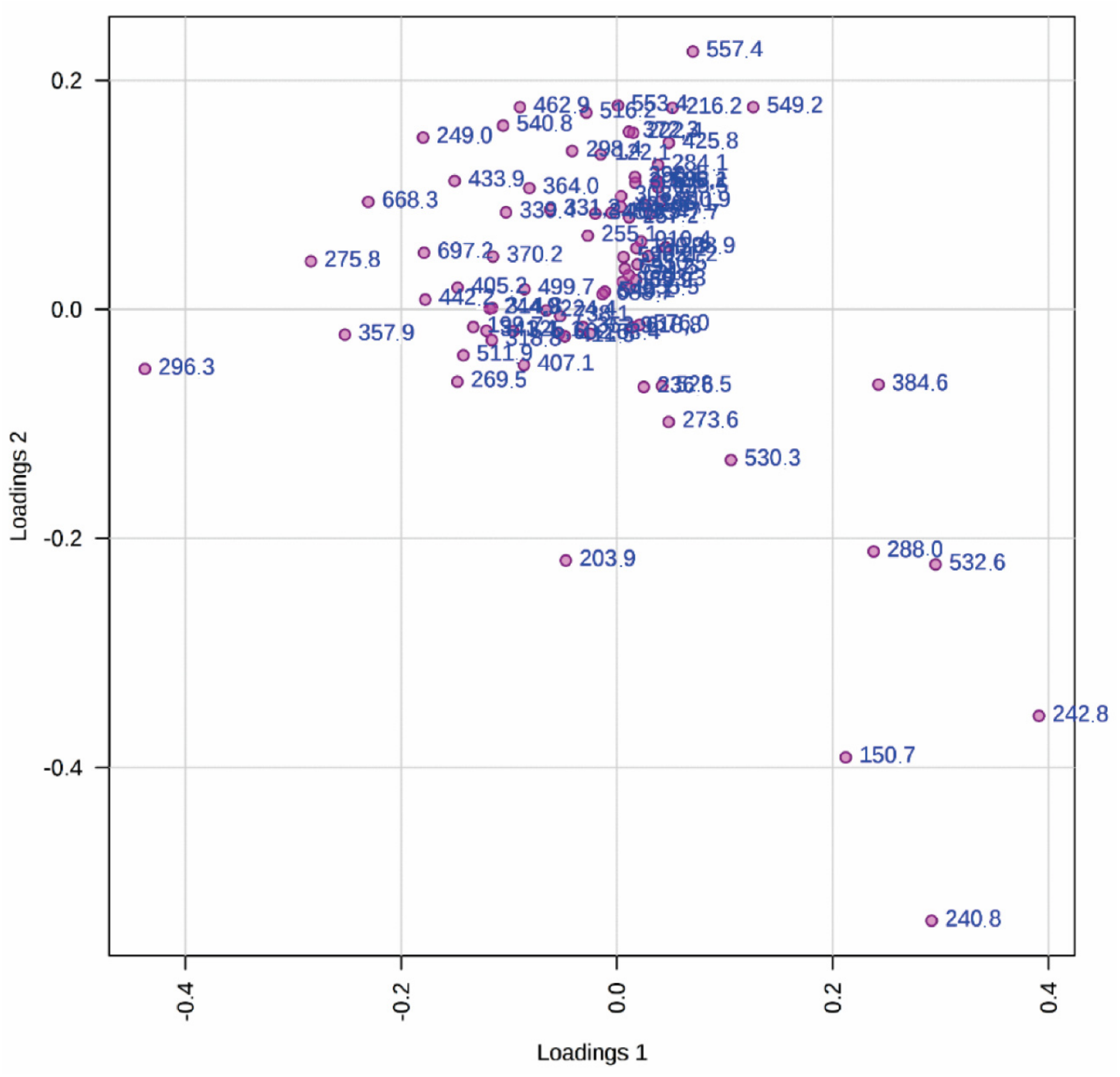

Figure S10. PLS-DA loadings for samples obtained in negative mode.

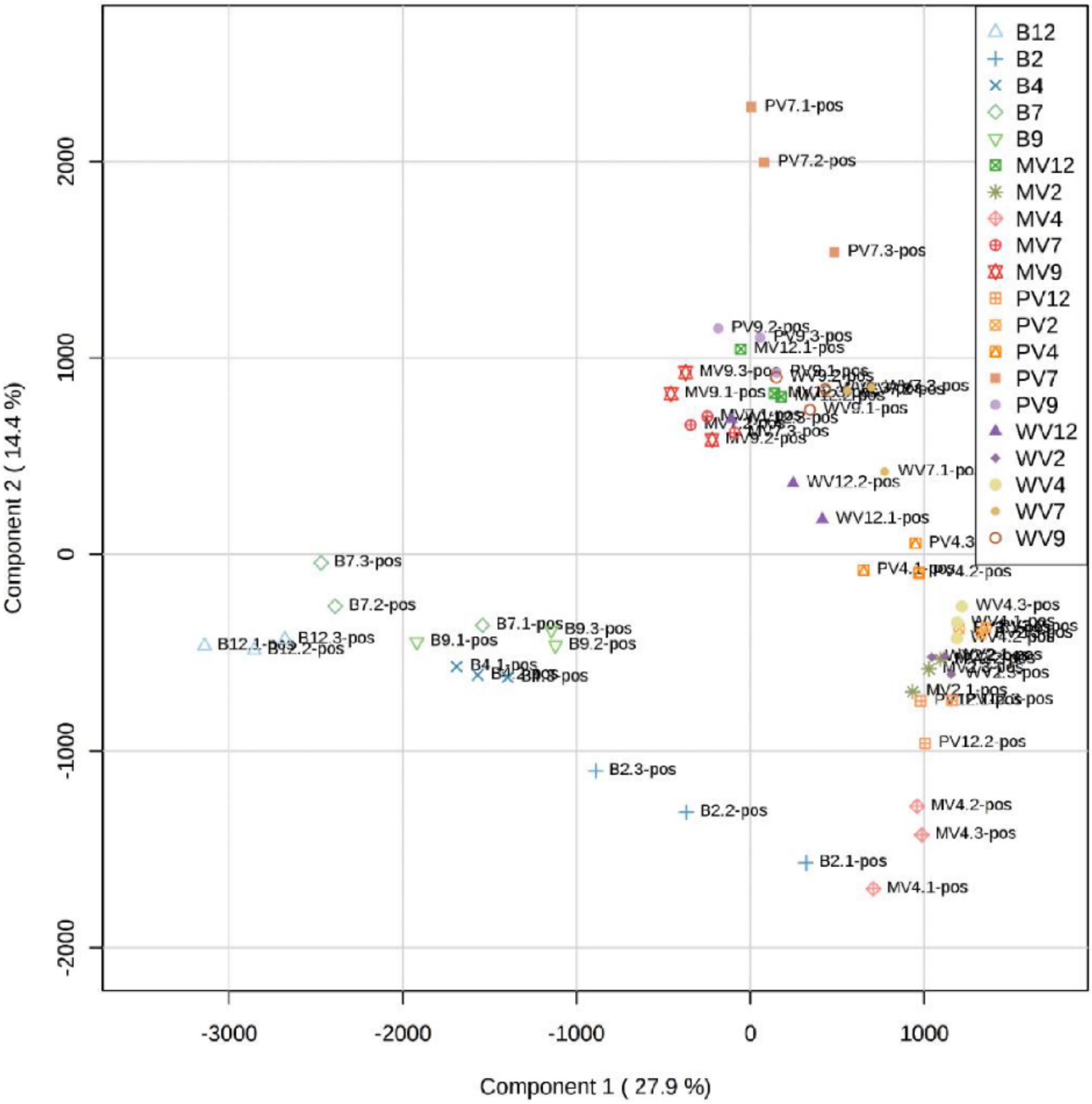

Figure S11. PLS-DA analysis of samples obtained in positive mode.

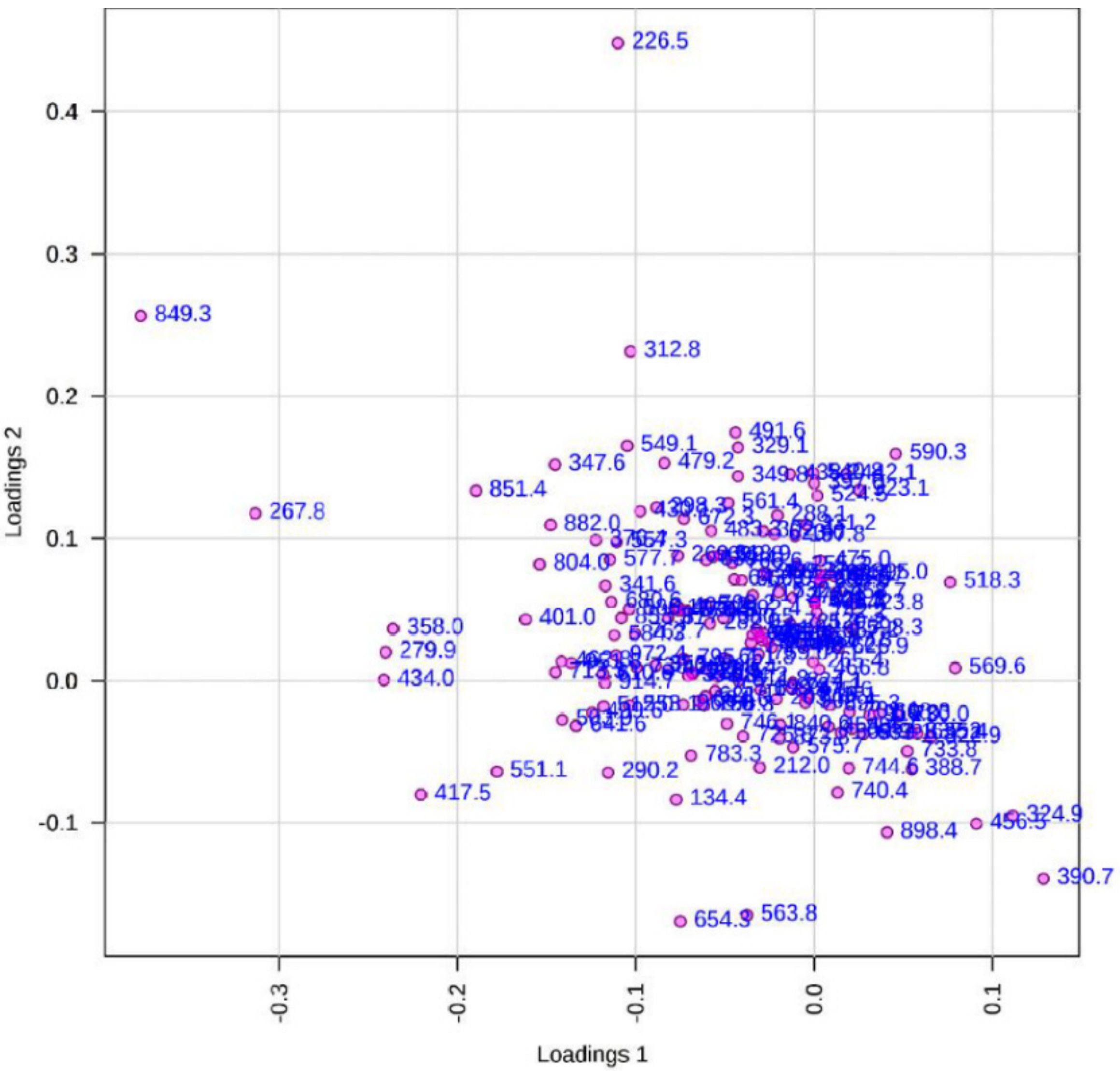

Figure S12. PLS-DA loadings for samples obtained in positive mode.

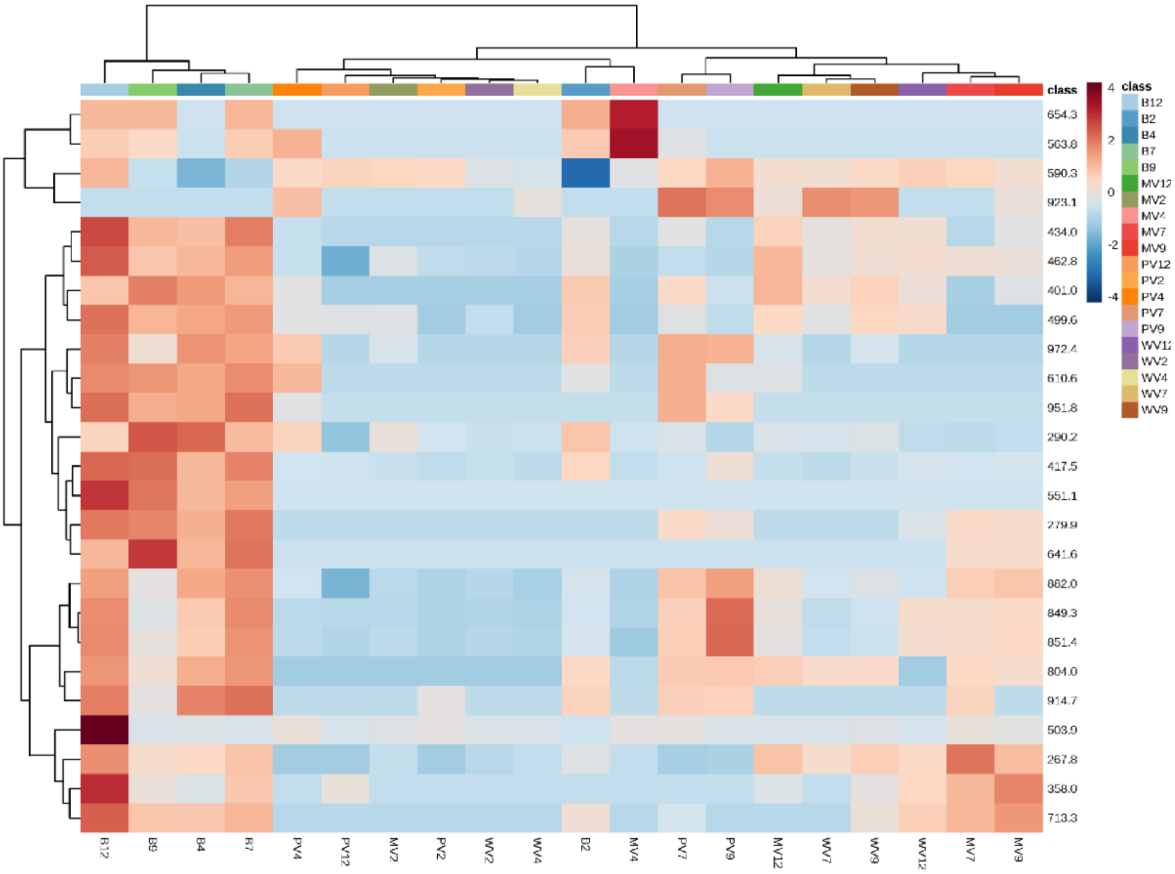

Figure S13. Heatmap for samples obtained in positive mode**.** The rows represent the top 25 VIP ions defined by PLS-DA. The columns refer to the means of normalized sample intensity. The greater the intensity for a detected ion, the redder the tone. In contrast, the smaller the intensity, the bluer the tone.

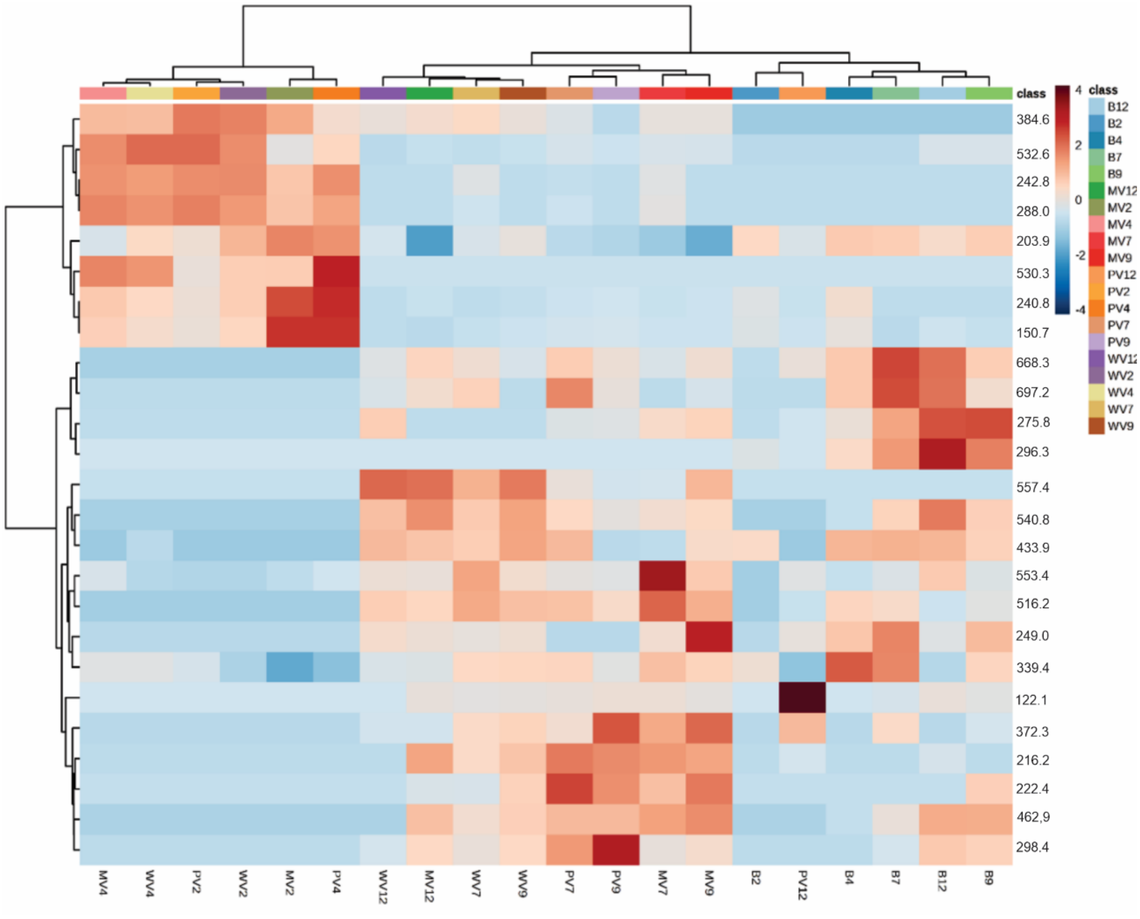

Figure S14. Heatmap for samples obtained in negative mode. The rows represent the top 25 VIP ions defined by PLS-DA. The columns refer to the means of normalized sample intensity. The greater the intensity for a detected ion, the redder the tone. In contrast, the smaller the intensity, the bluer the tone.

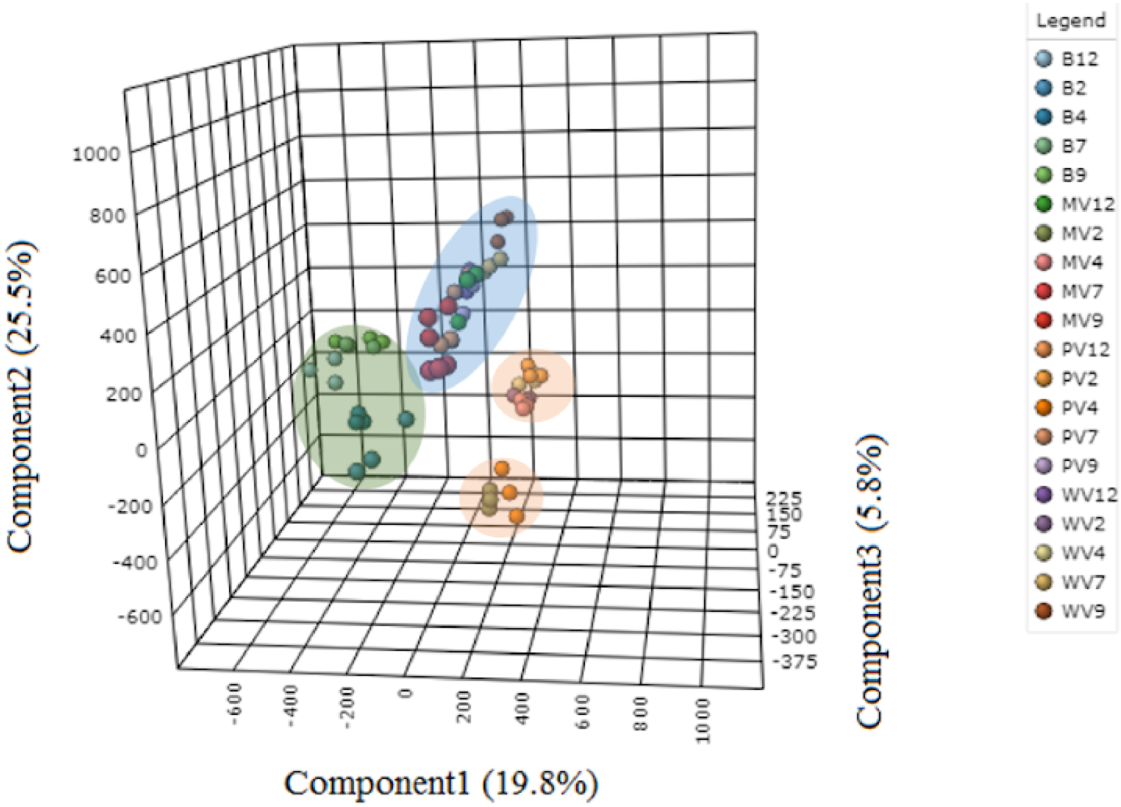

Figure S15. PLS-DA analysis of negative-mode data.

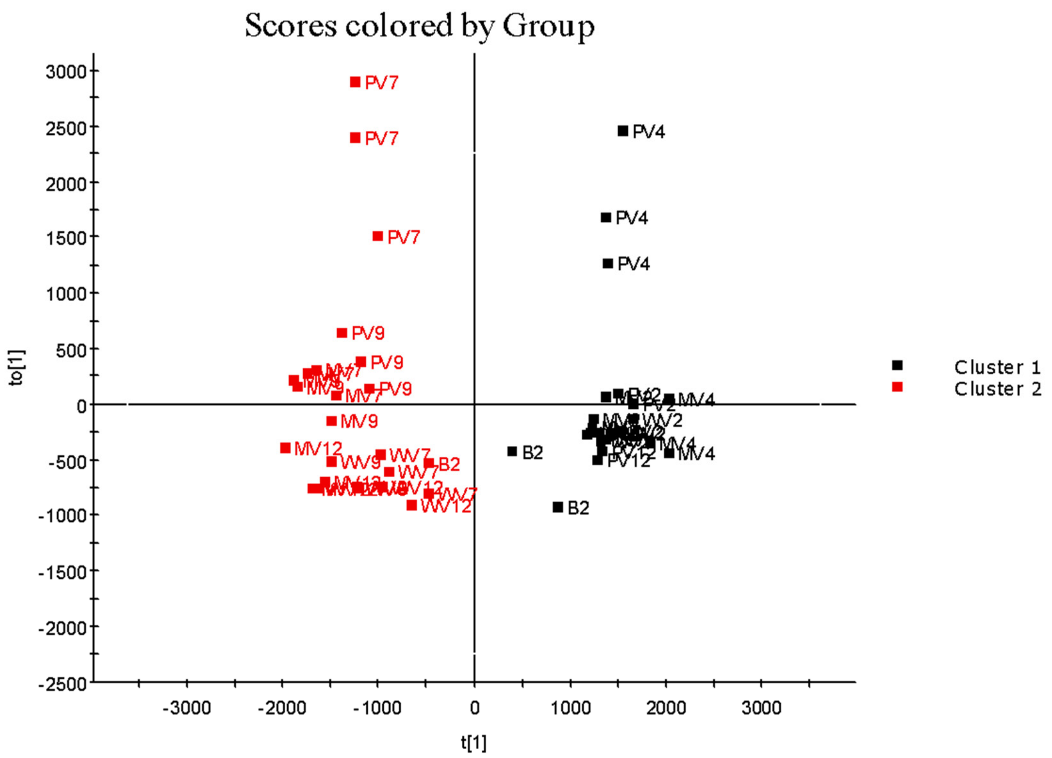

Figure S16. OPLS-DA analysis of positive-mode data.

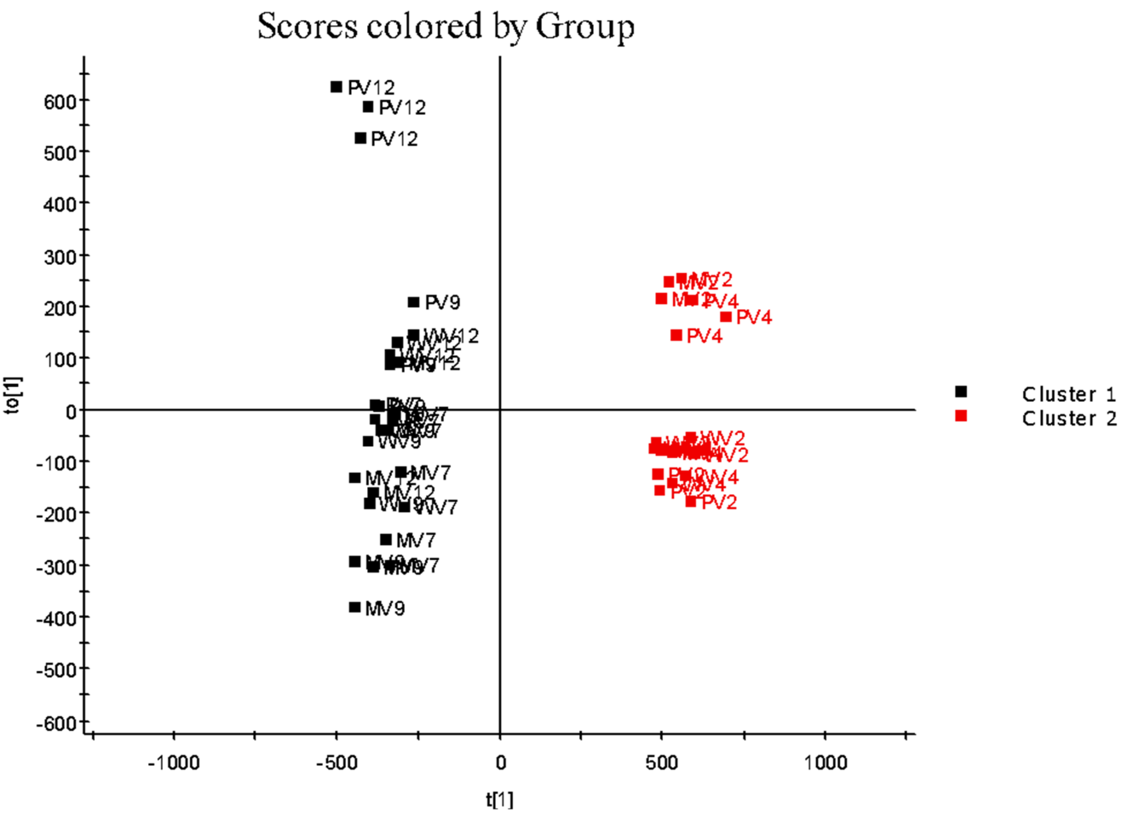

Figure S17. OPLS-DA analysis of negative-mode data.
